# D-serine, a novel uremic toxin, induces senescence in human renal tubular cells via GCN2 activation

**DOI:** 10.1038/s41598-017-11049-8

**Published:** 2017-09-11

**Authors:** Akira Okada, Masaomi Nangaku, Tzu-Ming Jao, Hiroshi Maekawa, Yu Ishimono, Takahisa Kawakami, Reiko Inagi

**Affiliations:** 1 Division of Nephrology and Endocrinology, The University of Tokyo Graduate School of Medicine, Tokyo, Japan; 2 Division of CKD Pathophysiology, The University of Tokyo Graduate School of Medicine, Tokyo, Japan

## Abstract

The prevalence of chronic kidney disease (CKD), characterized by progressive renal dysfunction with tubulointerstitial fibrosis, is increasing because of societal aging. Uremic toxins, accumulated during renal dysfunction, cause kidney damage, leading to renal deterioration. A recent metabolomic analysis revealed that plasma D-serine accumulation is associated with faster progression of renal dysfunction in CKD patients. However, the causal relationship and the underlying mechanisms remain unclear. Herein, we demonstrated that D-serine markedly induced cellular senescence and apoptosis in a human proximal tubular cell line, HK-2, and primary culture of human renal tubular cells. The former was accompanied by G2/M cell cycle arrest and senescence-associated secretory phenotype, including pro-fibrotic and pro-inflammatory factors, contributing to tubulointerstitial fibrosis. Integrated stress response mediated by the general control nonderepressible 2 played an important role in D-serine-induced tubular cell toxicity and pro-fibrotic phenotypes, accelerating CKD progression and kidney aging. D-serine upregulated the L-serine synthesis pathway. Furthermore, D-serine-induced suppression of tubular cell proliferation was ameliorated by L-serine administration, indicating that D-serine exposure induced an L-serine-deprived state in tubular cells, compensated by L-serine synthesis. Thus, this study unveils molecular mechanisms underlying D-serine-induced tubular damage and pro-fibrotic phenotypes, suggesting that D-serine is a uremic toxin involved in CKD pathogenesis.

## Introduction

Chronic kidney disease (CKD) is a common disease worldwide, often accompanied with progressive renal dysfunction^1^. It increases the risk of end-stage kidney failure, cardiovascular disease, and even premature death^2^. The cost of CKD is a burden for patients and the society^3^. However, no definitive treatment is currently available to overcome this problem. Researchers have been investigating the pathophysiology of CKD worldwide, and over the past two decades, “systems biology” approaches, ranging from genomics to metabolomics have been utilized in CKD research^4^. Recent technical development in the field of chiral amino acid metabolomics made it possible to distinguish between D- and L-amino acids and revealed the existence of D-amino acids in the living world, suggesting the possibility of studying cell biology based on amino acid chirality^5^. However, the biological functions of D-amino acids or their relevance in CKD are not fully elucidated.

A previous report revealed that plasma D-serine levels are elevated in aged individuals and patients with CKD^6^, while another study reported that the risk of advanced progression to end-stage kidney disease was approximately 3-fold higher in patients with CKD who had the highest levels of plasma D-serine than that of those who had the lowest levels^7^. These reports emphasized the importance of further investigation to understand CKD pathophysiology based on amino acid chirality. Thus, we studied the link between D-amino acids and CKD to address the pathophysiological role of D-amino acids in kidney injury.

Uremic toxins are a group of compounds that accumulate in proportion to renal dysfunction and exert deleterious effects on cells throughout the body. Some of them negatively affect cells and tissues in CKD, accelerate renal injury, and promote the progression of CKD^8^, thus creating a vicious cycle. D-serine accumulates in proportion to renal dysfunction. Thus, it is a novel uremic toxin if it harms cells or tissues. Therefore, we investigated D-serine biological functions to understand CKD pathophysiology from a novel viewpoint, amino acid chirality.

Amino acids are very important for biological functions, especially in stress signals. Depletion of some L-amino acids induces stress signals by mainly activating the general control nonderepressible 2 (GCN2)^9^. GCN2 is one of the four eukaryotic translation initiation factor 2 alpha (eIF2a) kinases, the other three being the double-stranded RNA-dependent protein kinase (PKR), the heme-regulated eIF2a kinase (HRI), and the PKR-like ER kinase (PERK). They converge on the eIF2a phosphorylation to activate the integrated stress response (ISR), which induces the expression of activating transcription factor 4 (ATF4), resulting in cell cycle and apoptosis-related signals such as C/EBP homologous protein (CHOP)^10^. The PERK-dependent ISR, via ATF4 and CHOP, induces the production of pro-inflammatory cytokines and the upregulation of p21 ﻿in﻿ human renal tubular cells, causing CKD progression^8^. The GCN2-dependent ISR, which is activated in response to L-amino acid starvation, also contributes to disease progression. It has been reported to aggravate pressure overload–induced congestive heart failure^11^. Therefore, in the present study, we hypothesized that the GCN2-dependent ISR may have a pathophysiological effect on human renal tubular cells.

CKD has been associated with cellular senescence^12^. In particular, tubular cell cycle arrest is closely linked to tubular senescence, leading to CKD progression^13^. Of note, recent evidence highlighted the fact that cellular senescence can induce senescence-associated secretory phenotype (SASP), which includes cell cycle arrest and secretion of pro-inflammatory cytokines and pro-fibrotic factors^14^. Thus, SASP can also be involved in senescence-associated tubular damage in CKD and be implicated in CKD progression. Given that D-serine is a putative predictive marker of poor prognosis of patients with CKD, we hypothesized that D-serine induces tubular damage via SASP-associated acceleration of cellular senescence. In the present study, we examined D-serine-mediated toxicity in human proximal tubular cells and its molecular mechanisms; in particular, D-serine-induced stress signals were investigated.

## Results

### D-serine, but not L-serine, suppresses proliferation and induces apoptosis in human tubular cells

**Figure 1:**
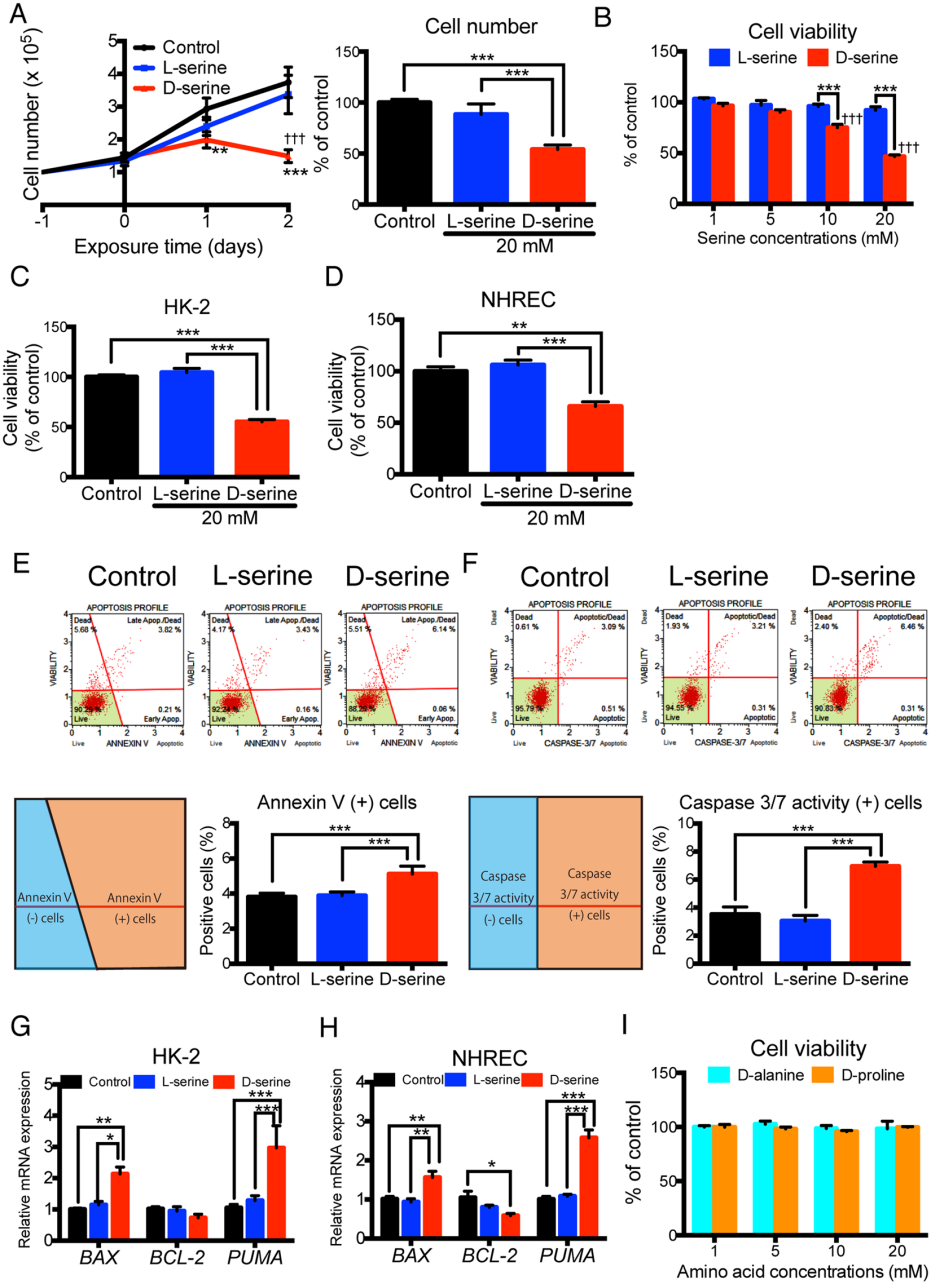
D-serine suppresses cellular proliferation and induces apoptosis in human tubular cells. (**A**) D-serine-containing medium significantly impaired proliferation at 24 h and 48 h when compared with the control medium. The cell numbers were expressed as relative numbers compared with the number on day −1 measured by the Muse Count & Viability Kit, which contains fluorescent dyes that distinguish live from dead cells. Each bar represents the average of control, 20 mM D-serine-treated, or 20 mM L-serine-treated cultures (n = 6). On the left graph, **P < 0.01, ***P < 0.001 compared to untreated control cells. ^†††^P < 0.001 compared to D-serine-treated cells. (**B**–**D**) At 10 and 20 mM, D-serine significantly decreased cell viability (**B**). At D-serine 20 mM, the cell viability of both HK-2 cells and normal human renal epithelial cells (NHREC) was decreased (**C**,**D**). HK-2 cells (**B**,**C**) or NHREC (**D**) were treated with L- or D-serine at 1–20 mM (**B**) or 20 mM (**C**,**D**). Viability was assessed by using MTS (n = 4 cultures for each treatment group). (**E**) HK-2 cells treated with 20 mM D-serine for 48 h showed a significant increase in Annexin V staining compared with that of control cells and cells treated with 20 mM L-serine. Apoptosis was induced by D-serine in HK-2 cells as shown by Annexin-V binding and 7-Amino actinomycin D (7-AAD) staining (n = 3 cultures per treatment group). (**F**) Increased activation of caspase 3/7 by D-serine, but not by L-serine, was confirmed (n = 3 cultures per treatment group). (**G**,**H**) Increased expression of pro-apoptotic *BAX* and *PUMA* in HK-2 cells (**G**) and NHREC (**H**), and decreased expression of anti-apoptotic *BCL-2* in NHREC (**H**) treated with D-serine for 48 h (n = 6 cultures per treatment group). (**I**) HK-2 cells were treated with D-alanine or D-proline at 1–20 mM for 48 h and viability was assessed by using MTS, with no significant changes with either amino acid added (n = 4 cultures for each treatment group). *P < 0.05, **P < 0.01, ***P < 0.001. All data are expressed as mean ± SEM.

To assess the pathophysiological effects of D-serine on human tubular cells, we first investigated the effect of D-serine on the proliferation rates of an immortalized human proximal tubular cell line, HK-2, and normal human renal epithelial cells (NHREC). When these cells were treated with various doses (0–20 mM) of D- or L-serine for 24 or 48 h, D-serine, but not L-serine, reduced the cell proliferation rate in a dose-dependent manner, as measured by a cell count assay and MTS assay (Fig. 1A–D). Furthermore, Annexin V staining and caspase 3/7 activity analysis revealed that D-serine (20 mM for 48 h) induced more apoptosis in HK-2 cells than in controls, while L-serine showed no such apoptotic effect (Fig. 1E,F). This tubular cell apoptosis after D-serine treatment was associated with increased expression of the pro-apoptotic genes, *BAX* and the p53-upregulated modulator of apoptosis (*PUMA*) in both HK-2 cells and NHREC, and decreased expression of the anti-apoptotic gene, *BCL-2*, in NHREC, although *BCL-2* expression only tended to decrease in HK-2 cells (Fig. 1G,H). These results demonstrated that D-serine induced mitochondria-dependent apoptosis, resulting in a reduction of viable tubular cells. Such tubular toxicity was not observed with other D-amino acids such as D-alanine and D-proline, both of which also accumulate during kidney dysfunction (Fig. 1I).

### D-serine induces G2/M cell cycle arrest in human tubular cells

**Figure 2:**
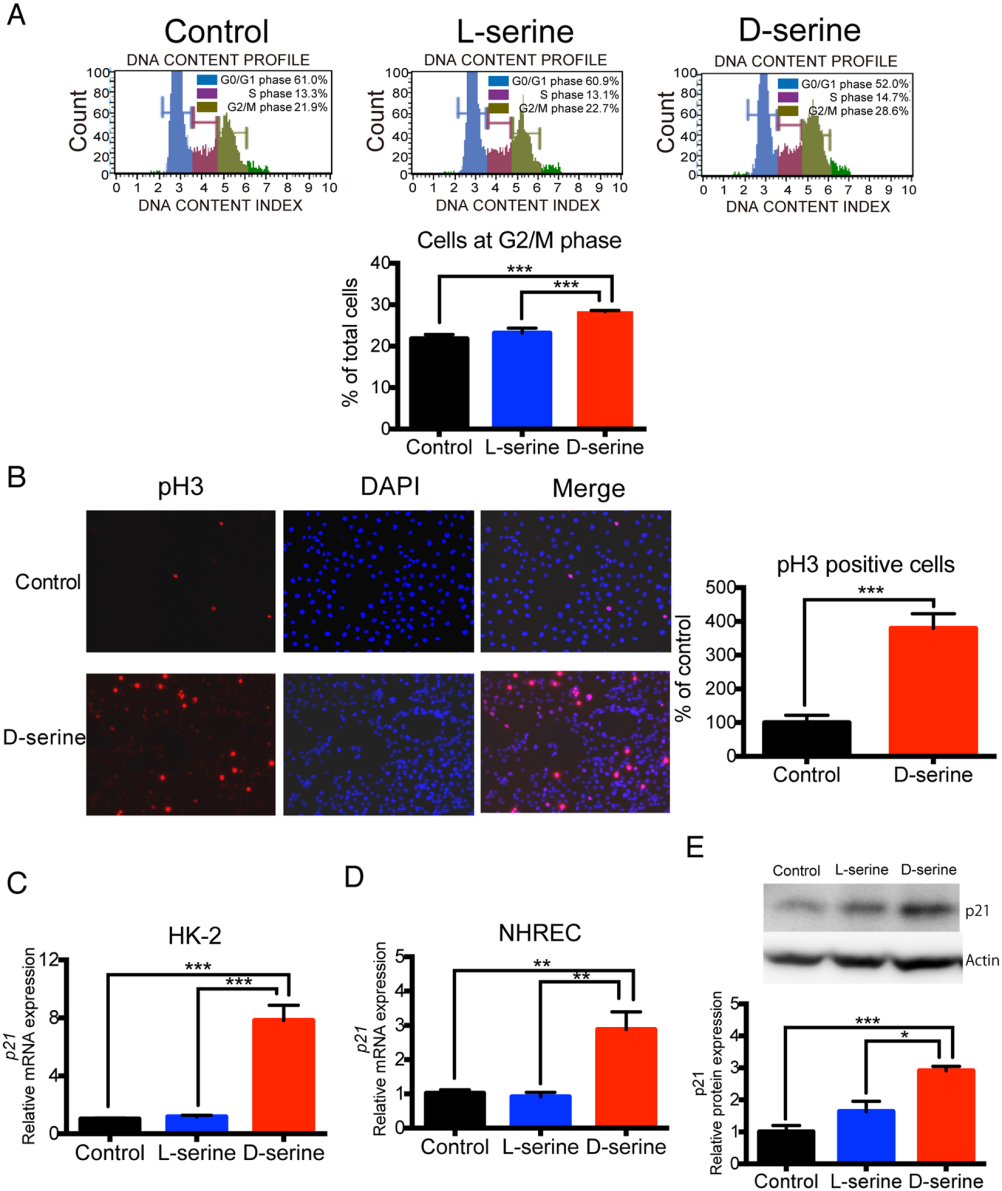
D-serine induces cell cycle arrest at the G2/M phase. (**A**) Cell cycle analyses by PI staining after 48 h of treatment with L- or D-serine indicated that D-serine induced cell cycle arrest at G2/M (n = 3 cultures per treatment group). (**B**) Representative images of immunofluorescence staining of phosphorylated histone H3 (pH3). D-serine increased pH3-positive HK-2 cells. Quantification of the percentage of cells positive for pH3 in the absence or presence of 20 mM D-serine (n = 7 areas chosen randomly per treatment group). (**C**,**D**) Upregulation of *p21* in HK-2 cells (**C**) and NHREC (**D**) treated with 20 mM D-serine for 48 h as determined by qRT-PCR (n = 6 cultures per treatment group). (**E**) Western blot for p21 expression. Increased expression of p21 was confirmed at the protein level in HK-2 cells (n = 3 cultures per treatment group). A typical image and densitometric analysis are shown. *P < 0.05, **P < 0.01, ***P < 0.001. All data are expressed as mean ± SEM.

The reduction in the cell proliferation rate by D-serine was greater than the increase in the proportion of apoptotic cells (Fig. 1A,E,F), suggesting that D-serine mediated tubular cell arrest. Indeed, a propidium iodide (PI)-based cell cycle assay revealed that D-serine induced cell cycle arrest at the G2/M phase in HK-2 cells, whereas L-serine did not (Fig. 2A). Phosphorylation of histone H3, which is a representative G2/M marker^15^, was also significantly increased in D-serine-treated HK-2 cells (Fig. 2B). To confirm these results, we compared the expression of *p21*, a cell cycle inhibitor, in tubular cells with or without D-serine by real-time PCR and western blot, and confirmed that *p21* was upregulated by D-serine in these cells at the mRNA and protein levels (Fig. 2C–E).

### D-serine induces tubular senescence with the SASP

**Figure 3:**
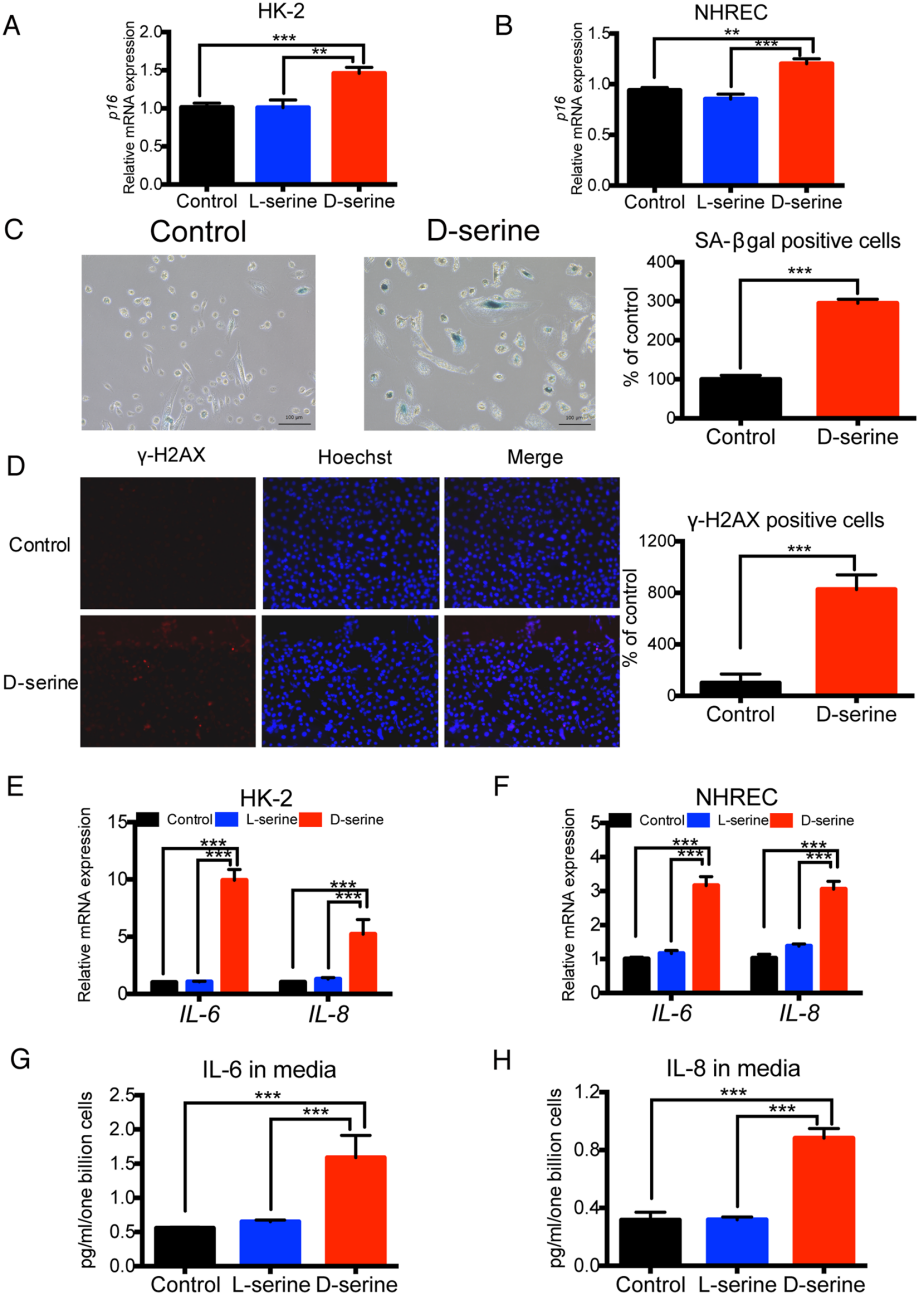
D-serine induces tubular senescence with senescence-associated secretory phenotype (SASP). (**A**,**B**) Upregulation of *p16* gene expression in HK-2 cells (**A**) and NHREC (**B**) treated with 20 mM D-serine for 48 h (n = 6 cultures per group). (**C**) Representative pictures of SA-β gal staining of control and D-serine treated NHREC and quantitative assessment. D-serine increased NHREC positive for SA-β gal staining (n = 7 areas chosen randomly per treatment group). (**D**) Representative pictures of immunofluorescence staining of γ-H2AX of control and D-serine treated HK-2 cells and quantitative assessment. D-serine increased HK-2 cells positive for γ-H2AX staining (n = 7 areas chosen randomly per treatment group). (**E**,**F**) Expression levels of a pro-inflammatory cytokine, *IL-6*, and chemokine, *IL-8*, two SASP markers, were upregulated in HK-2 cells (**E**) and NHREC (**F**) by 20 mM D-serine treatment for 48 h (n = 6 cultures per group). (**G**,**H**) D-serine also induced the secretion of IL-6 (**G**) and IL-8 (**H**) into the medium as measured by chemiluminescence enzyme-linked immunosorbent assay (ELISA) (**G**, n = 3 cultures per group) and homogeneous time resolved fluorescence (HTRF), respectively (**H**, n = 3 cultures per group) in HK-2 cells. **P < 0.01, ***P < 0.001. All data are expressed as mean ± SEM.

Previous studies reported that, when arrested at G2/M, injured renal tubular cells adopt a pro-fibrotic phenotype, which influences surrounding epithelial cells^13, 16^. After confirming that D-serine caused tubular cell cycle arrest at G2/M, we investigated the fate of arrested cells. First, senescence markers involved in cell cycle inhibition, including *p16* and *p21* (see Fig. 2C–E), were increased in D-serine-treated tubular cells (Fig. 3A,B). Furthermore, as expected, the D-serine-treated tubular cells showed a senescence phenotype, which was estimated by cellular senescence markers such as senescence-associated beta-galactosidase (SA-β gal) and γ–H2AX (Fig. 3C,D). Importantly, the D-serine-induced tubular cell senescence was associated with SASP in HK-2 cells and NHREC. The expression of representative SASP molecules, including pro-inflammatory cytokine, IL-6, and chemokine, IL-8, were increased with D-serine treatment (Fig. 3E–H). These data demonstrated that D-serine induced tubular cell senescence accompanied with SASP.

### D-serine-induced tubular cell senescence is mediated via GCN2 activation as an integrated stress response

**Figure 4:**
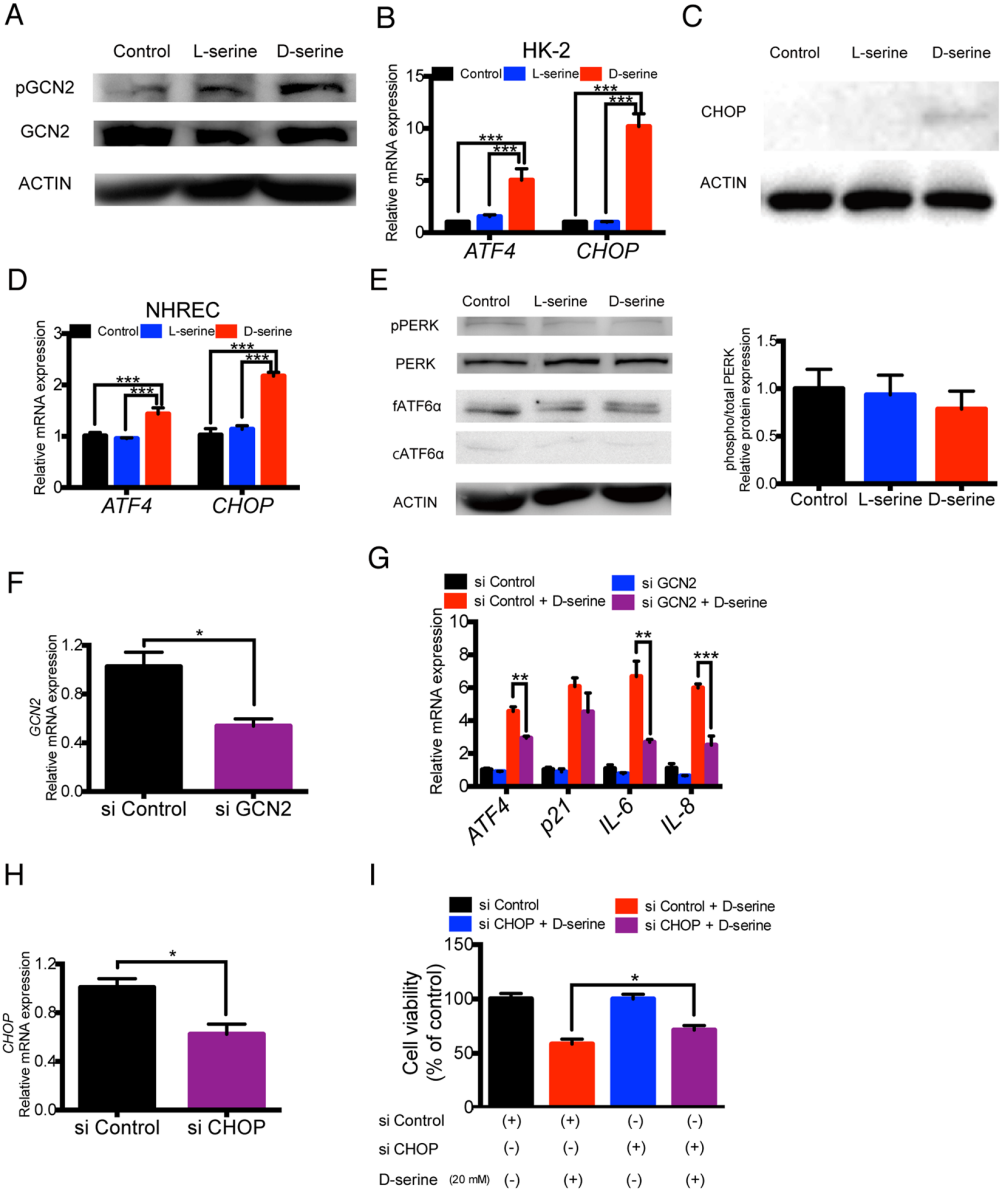
D-serine induces tubular cell senescence via GCN2 activation as an integrated stress response. (**A**) D-serine (20 mM for 48 h) phosphorylated GCN2 in HK-2 cells. (**B**) D-serine also upregulated the ISR signalling molecules, *ATF4* and *CHOP*, in HK-2 cells (n = 6 cultures per treatment group). (**C**) D-serine exposure increased CHOP protein expression compared with that in control and L-serine-treated HK-2 cells. (**D**) D-serine exposure (20 mM for 48 h) upregulated ISR-related molecules in NHREC (n = 6 cultures per group). (**E**) Western blotting analysis showed no change in PERK, phosphorylated PERK (pPERK), or cleaved ATF6α expression in D-serine-treated HK-2 cells. Especially, pPERK, an upstream molecule of ATF4, showed no significant increase with D-serine treatment (n = 4 cultures per treatment group). (**F**) siRNA-mediated GCN2 knockdown induced *GCN2* downregulation by approximately 50% 48 h after transfection in HK-2 cells (n = 4 cultures per treatment group). (**G**) siRNA-mediated GCN2 knockdown suppressed the upregulation of *ATF4*, *IL-6*, and *IL-8* in D-serine-treated HK-2 cells (n = 4 cultures per treatment group). (**H**) siRNA-mediated CHOP knockdown induced *CHOP* downregulation by approximately 40% 48 h after transfection in HK-2 cells (n = 4 cultures per treatment group). (**I**) siRNA-mediated CHOP knockdown ameliorated the cell proliferation impairment in HK-2 cells using MTS (n = 4 cultures per treatment group). *P < 0.05, **P < 0.01, ***P < 0.001. All data are expressed as mean ± SEM.

Next, we hypothesized that exposure to D-serine may disrupt amino acid status associated with the activation of ISR, leading to tubular cell senescence. To address this issue, we examined the association between D-serine and GCN2, which is activated by L-amino acid starvation and plays key roles in modulating amino acid metabolic pathways and ISR as a serine/threonine-protein kinase. Indeed, D-serine activated GCN2 in HK-2 cells, as evidenced by the increase in phosphorylation compared to the controls or L-serine-treated cells (Fig. 4A). Importantly, GCN2 activation by D-serine induced the downstream ISR signalling pathway; *ATF4* and *CHOP* were upregulated at the mRNA level (Fig. 4B) and CHOP was increased at the protein level as well (Fig. 4C) in D-serine-treated HK-2 cells. A similar pattern of GCN2-mediated ISR activation was also observed in NHREC (Fig. 4D).

Because CHOP is also known as an effector molecule of ER stress, which is related to ISR, we examined whether other ER stress modulators, PERK and ATF6α, were also involved in the D-serine-induced ISR. However, PERK and ATF6α were not activated by phosphorylation and cleavage, respectively (Fig. 4E).

To assess the mechanistic contribution of GCN2 activation in tubular cell senescence, we used siRNA transfection to knock down GCN2. GCN2 knockdown (Fig. 4F) significantly mitigated the D-serine-induced activation of ISR, as demonstrated by the expression level of *ATF4* (Fig. 4G). D-serine-induced SASP was also ameliorated by GCN2 knockdown; D-serine-induced upregulation of *IL-6* and *IL-8* was markedly decreased, while the expression of *p21* tended to decrease in the tubular cells after GCN2 knockdown (Fig. 4G). When CHOP, which is activated by GCN2-mediated ISR pathway, was knocked down by siRNA (Fig. 4H), and D-serine-induced impairment in cell proliferation was significantly ameliorated (Fig. 4I). These data demonstrated that D-serine-induced GCN2-mediated ISR caused SASP and impaired cell proliferation of tubular cells.

### D-serine activates the L-serine synthesis pathway and D-serine-induced tubular toxicity is counteracted by L-serine

**Figure 5:**
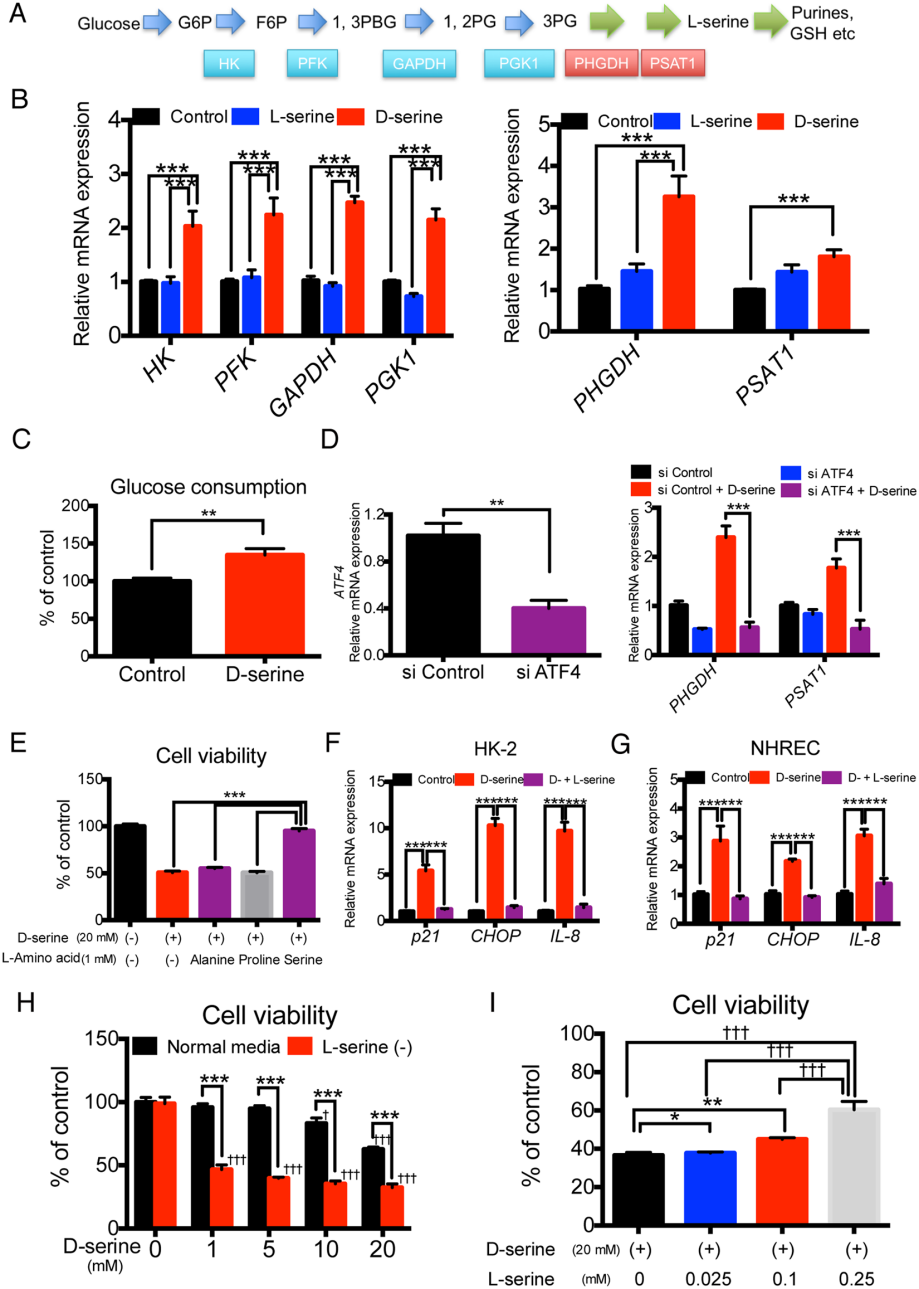
D-serine activates the L-serine synthesis pathway and D-serine-induced tubular toxicity is altered by L-serine concentrations in D-serine-containing medium. (**A**) The L-serine synthesis pathway from glucose, with important enzymes. G6P; glucose 6-phosphate, F6P; fructose 6-phosphate, 1,3PBG; 1,3-bisphosphoglyceric acid, 3-PG; 3-phosphoglycerate, GSH; glutathione. (**B**) The expression of enzymes involved in the L-serine synthesis pathway was upregulated at the mRNA level by D-serine (20 mM for 48 h) in HK-2 cells (n = 6 cultures per treatment group). (**C**) Glucose consumption was enhanced by 20 mM D-serine (n = 3 cultures per treatment group). (**D**) siRNA-mediated ATF4 knockdown induced *ATF4* downregulation by approximately 60% 48 h after transfection in HK-2 cells. siRNA-mediated ATF4 knockdown suppressed the upregulation of *PHGDH* and *PSAT1* in D-serine-treated HK-2 cells (n = 4 cultures per treatment group). (**E**) L-serine (1 mM) completely reversed the reduction of HK-2 cell viability induced by 20 mM D-serine, while L-alanine or L-proline did not (n = 4 cultures per treatment group). (**F**,**G**) L-serine (1 mM) completely reversed the increased expression of *p21*, *CHOP*, and *IL-8* induced by 20 mM D-serine in HK-2 cells (**F**) and NHREC (**G**) (n = 4 cultures per treatment group). (**H**) As shown by MTS assay, D-serine-induced cell toxicity (20 mM) was enhanced when using L-serine free medium in comparison with that when using normal medium (L-serine 0.25 mM) in HK-2 cells (n = 4 cultures per treatment group). ^†††^P < 0.001 compared to control cells. (**I**) D-serine cell toxicity increased in proportion with decreased L-serine addition into the medium containing 20 mM D-serine (n = 4 cultures per treatment group). ^†^P < 0.05 compared with control cells, ^†††^P < 0.001 compared with control cells. *P < 0.05, **P < 0.01, ***P < 0.001. All data are expressed as mean ± SEM.

The regulation of the L-serine synthesis pathway (Fig. 5A) has recently garnered the attention in the field of cancer research, because L-serine starvation upregulates the senescence marker, p21 and the GCN2-dependent ISR^17^, thus promoting its downstream signals, leading to apoptosis and cell cycle arrest. Considering that D-serine activated GCN2, which is a sensor of L-amino acid deficiency, we examined the effects of D-serine on the L-serine synthesis pathway. First, we assessed the expression levels of enzymes involved in the synthesis of L-serine from glucose. Hexokinase (*HK*), phosphofructokinase 1 (*PFK1*), glyceraldehyde-3-phosphate dehydrogenase (*GAPDH*), and phosphoglycerate kinase 1 (*PGK1*), which supplies L-serine precursors from glucose, and L-serine synthesis pathway enzymes, phosphoglycerate dehydrogenase (*PHGDH*) and phosphohydroxythreonine aminotransferase 1 (*PSAT1*), were all significantly upregulated after 48 h of D-serine treatment (Fig. 5B). Furthermore, D-serine slightly, but significantly, augmented glucose consumption, suggesting the increased flow from glucose to L-serine (Fig. 5C). Since PHGDH and PSAT1 are essential enzymes for L-serine synthesis known to be regulated by ATF4^18–20^ and D-serine upregulated *ATF4* via GCN2 activation (Fig. 4G), we knocked down ATF4 to confirm that GCN2-mediated ISR induced the L-serine synthesis pathway. As expected, ATF4 knockdown suppressed the upregulation of both *PHGDH* and *PSAT1* genes by D-serine treatment (Fig. 5D).

The fact that D-serine activates the L-serine pathway raises two possibilities about the mechanisms by which D-serine mitigates tubular cell proliferation. One is that increased intracellular D-serine contributes to the suppression of cell proliferation by itself. The other is that D-serine deranges the L-amino acid sensing status, L-serine “pseudo”-deprivation, and activates its sensor GCN2, leading to cellular senescence. To determine which holds true, we examined the effects of D-serine with addition or deprivation of L-serine. Surprisingly, the addition of no more than 1 mM L-serine completely reversed the D-serine-induced decrease in HK-2 cell numbers (Fig. 5E). In contrast, the addition of other L-amino acids did not ameliorate the effects of D-serine (Fig. 5E), suggesting that this ability to reverse D-serine toxicity was not generic for L-amino acids, but specific to L-serine. The increased expression of effector molecules such as *p21*, *CHOP*, and *IL-8* induced by D-serine treatment were also reversed by treatment with 1 mM L-serine in both HK-2 cells and NHREC (Fig. 5F and G). On the other hand, when L-serine was depleted in the medium, the D-serine-induced toxicity was augmented in HK-2 cells (Fig. 5H; and when comparing the D-serine dose–responses in Fig. 1A to that in Fig. 5H). Furthermore, L-serine addition into the medium containing 20 mM D-serine mitigated the deleterious effects of D-serine on cell survival in a dose-dependent manner (Fig. 5I). These findings suggest that D-serine activates the L-serine synthesis pathway and that the proportion of D-/L-serine is critical for D-serine toxicity to tubular cells.

## Discussion

**Figure 6:**
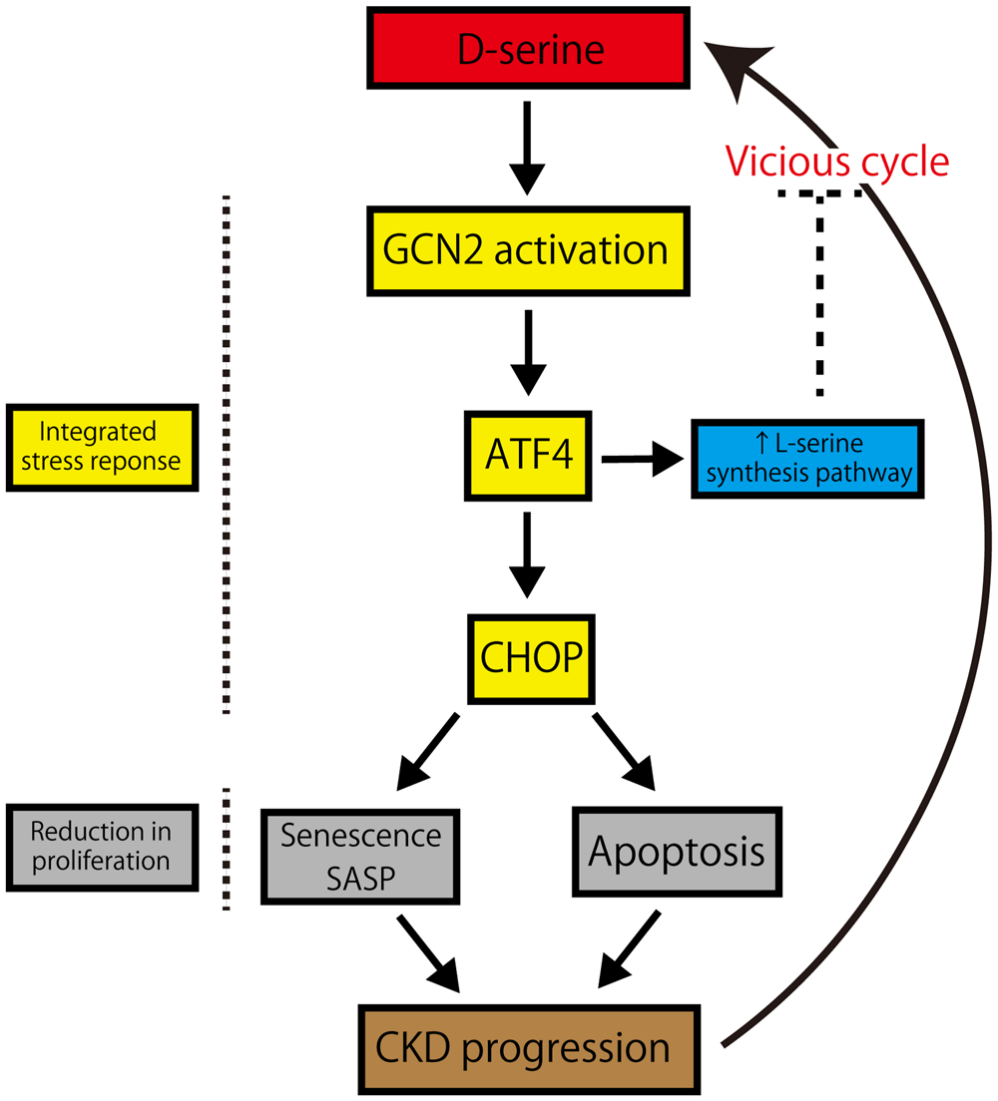
D-serine accelerates CKD via activation of GCN2-mediated ISR. High concentrations of D-serine activate GCN2-mediated ISR, which upregulates ATF4 and CHOP, resulting in cell cycle arrest and apoptosis. Tubular cell senescence, SASP, and apoptosis lead to CKD progression. With increased CKD severity, plasma D-serine concentration rises, creating a vicious cycle of progressively greater D-serine toxicity and renal failure. On the other hand, ATF4 up-regulates L-serine synthesis response, which may partially contribute to the amelioration of the “pseudo-” L-serine starvation state. Consistently, L-serine supplementation abrogated cellular toxicity induced by D-serine.

In this study, we demonstrated that a novel uremic toxin, D-serine, has highly deleterious effects on human renal proximal tubular cells *in vitro*. Exposure to D-serine led to cellular senescence with G2/M cell cycle arrest and SASP, accompanied with enhanced apoptosis. Furthermore, the D-serine-induced tubular cell toxicity upregulated pro-fibrotic factors, possibly promoting tubulointerstitial fibrosis. This was initiated by the activation of GCN2, a sensor of amino acid status and ISR initiator^17, 21, 22^. Low concentrations of serum D-serine are normally maintained by renal excretion^23^. It is suggested that D-serine toxicity augments kidney dysfunction and thus increases D-serine levels, creating a vicious cycle, which leads to progressively greater kidney senescence and nephrotoxicity (Fig. 6), ultimately resulting in CKD progression.

In this report, we examined a connection between D-serine and CKD progression. D-serine was used in clinical trials for the treatment of schizophrenia without any reports of renal complications^24–28^. However, one study reported proteinuria without renal dysfunction in a patient who received high doses of D-serine^29^, suggesting the possibility of dose-dependent toxicity of D-serine to the human kidney. Our study supported a possible nephrotoxicity by D-serine. Meanwhile, to address the selectivity of D-serine toxicity in other cell types, we tested the effect of D-serine (20 mM) on human cells such as hepatocellular carcinoma (HepG2) or primary vein endothelial cells (HUVEC) as well as mouse mesangial cell line (SV40 MES 13). The results showed that D-serine did not affect the cell viability in these cell types, suggesting that the selectivity of D-serine toxicity seems to depend on the cell type, but not on D-amino acid oxidase (*DAO*) expression (Supplementary Fig. 1A,B). Although further investigation is necessary to evaluate D-serine nephrotoxicity in humans more precisely, our study may shed light on novel pathophysiological function of D-serine as a senescence inducer.

We found toxicity of D-serine, but not of other D-amino acids, in human renal tubular cells. Likewise, accumulating evidence suggests different effects of different D-amino acids in the different organs. For example, in the field of ophthalmology, abnormal proteins rich in advanced glycation end-products and D-β-aspartic acid co-localize in the amyloid lesions in gelatinous drop-like corneal dystrophy^30^, and D-β-aspartic acid-containing proteins were found in samples with pterygium, but not in samples without pterygium^31^. In addition, in the field of endocrinology, D-alanine is co-localized with beta cells in the pancreas, and with ACTH-producing cells in the anterior pituitary gland^32^. Another study has shown that the D-alanine content in pancreas islets is affected by glucose stimulation^33^, suggesting that D-alanine may be related to glucose control in the plasma. This accumulated evidence may highlight the possibility of the tubular cell-specific D-serine toxicity. Future studies are required to clarify the different pathophysiological function between D-serine and other D-amino acids in tubular cells.

The activation of GCN2, usually provoked by L-amino acid starvation, causes the upregulation of ATF4 and CHOP, leading to cell cycle arrest^34^. This set of responses is called the ISR. The ISR converges ATF4 activation with activation of GCN2, PKR, HRI, or PERK^10^. GCN2 initially senses an increase in uncharged tRNA, which is considered a signal of L-amino acid starvation. Most studies on GCN2 activation by L-serine starvation focused on cancer cells^14^ or fibroblasts^35^, but not on cells in mature and healthy organs. In cancer cells, the depletion of L-serine causes impaired cell proliferation via GCN2-dependent ISR^17^. However, when we cultured HK-2 cells without L-serine, no change in cell viability was observed (Fig. 5H). This difference between HK-2 and cancer cells may be attributed to the much higher level of metabolism in the latter. Nonetheless, we demonstrated that D-serine could activate GCN2 in healthy kidney tubular cells as well as in cancer cells.

PERK-dependent ISR can be ameliorated by the knockdown of effector molecules, especially CHOP, in human tubular cells^8^. In this study, GCN2-dependent ISR was ameliorated by the siRNA-mediated knockdown of CHOP, an important ISR effector molecule. This knockdown reversed the D-serine-induced reduction in the number of viable cells. GCN2 knockdown also reduced the expression of *ATF4* and SASP molecules, IL-6 and IL-8 (a pro-inflammatory cytokine and a chemokine, respectively), indicating that blocking the ISR can mitigate the toxic effects of D-serine on renal tubular cells. IL-6 and IL-8 are at the same time important SASP markers. Metabolic alteration stress causes ISR, accompanying IL-6 and IL-8 upregulation, and causing renal fibrosis^36^. Therefore, to modulate ISR may be a therapeutic candidate to slow down CKD progression via downregulation of SASP markers.

Prolonged exposure to D-serine upregulated the molecules involved in ISR, inducing cellular senescence with SASP and apoptosis. D-serine also concomitantly promoted the L-serine synthesis pathway (as evidenced by upregulation of multiple enzymes of the L-serine synthesis pathway and increased glucose consumption). Two possible scenarios can explain these phenomena: one involves D-serine-induced cell toxicity, while the other involves the tubular cells somehow being inaccessible to L-serine. Considering the fact that D-serine-induced tubular cell toxicity relates to the L-amino acid sensor, GCN2, and depends on the concentrations of D/L-serine in the medium and that the damage induced by D-serine was exaggerated by L-serine deficiency (Fig. 5H,I), the latter seems more probable.

This study has the following limitations. First, we only reported *in vitro* results using high concentrations of D-serine. While considering confirming our current findings by *in-vivo* study, we are very much concerned about CKD model animals we should use from two points of view. One is existence of species-specific unknown mechanisms of D-serine nephrotoxicity. It is reported that D-serine nephrotoxicity is caused by DAO in rats, while it is not in other mammals, such as mice, guinea pigs, or hamsters, all of which have similar levels of DAO activity with rats^37^. In fact, we utilized two kinds of human proximal tubular cells (HK-2 and NHREC) which do not express *DAO* (Supplementary Fig. 1A), and demonstrated DAO-independent and GCN2-dependent D-serine nephrotoxicity. The other is character of CKD model animal. The common CKD model in mice is induced by adenine diet, but adenine affects amino acid metabolism^38^. Taken all together, we are currently screening appropriate CKD models including diabetic rats and mice for future *in-vivo* studies to investigate the link between the change in D/L-serine ratio and tubular phenotypic changes. Secondly, the mechanism by which D-serine enters cells and is distributed within the cells remains unclear. While it is currently difficult to measure intracellular and extracellular D-serine, a feasible method has been recently developed^39^. In future studies, it would be important to directly demonstrate the accumulation of D-serine in organelles. Finally, the concentration of D-serine used in this study was higher than that found in the urine in subjects; in a normal human population, urinary D-serine concentrations range is approximately 150 µM^40^. From the fact that urinary concentration of the most uremic toxins is increased as CKD progresses^41^, urinary D-serine concentrations in CKD patients are supposed to be increased more than 150 µM. The urinary concentrations of representative uremic toxins, indoxyl sulfate and kynurenic acid, in CKD patients are increased by 41.3 and 27.6 times higher than those in healthy individuals, respectively^41^. In addition, the plasma D-serine levels of CKD patients are 5-fold to 10-fold higher than those of healthy population^23^, suggesting that urinary D-serine concentration of CKD patients can be estimated more than 1 mM. The evidence mentioned above may support the adequacy to use one order of magnitude higher D-serine concentration due to the short exposure period (2 days) in our *in-vitro* study (Fig. 1B). Our speculation is also supported by other findings showing that the same order of magnitude (1 mM) of D-serine concentration could induce tubular cell toxicity under certain conditions such as L-serine deprivation (Fig. 5H).

In conclusion, we provide evidence that D-serine induces senescence in renal tubular cells with pro-inflammatory and pro-fibrotic SASP, accompanied by apoptosis. This nephrotoxicity can contribute to CKD progression. Aggravated renal dysfunction due to D-serine-induced senescence or death of tubular cells can further increase systemic D-serine by its reduced excretion, resulting in progressive damage to tubular cells and kidneys. Furthermore, our findings indicated that higher D-serine concentrations lead to tubular cell injury, suggesting that the ratio can be utilized as a marker for progressive renal deterioration in patients with CKD. Thus, we proposed reducing the absolute concentration of D-serine, giving L-serine to subjects with CKD, and blocking the downstream effector mechanisms of ISR as potential therapeutic strategies against CKD.

## Methods

### Cell lines and cell culture

The human proximal tubular cell line, HK-2, was cultured in DMEM/F12 supplemented with 5% foetal calf serum (FCS). L-serine and D-serine (Wako Pure Chemical Industries, Osaka, Japan) were dissolved in very small amounts of distilled water and added directly to the medium before use. Normal human renal epithelial cells (NHREC) were purchased from Kurabo (Osaka, Japan) and cultured in Renalife medium (Lifeline Cell Technology, Frederick, MD, USA). The amino acids, D-/L-alanine, D-/L-serine, and D-/L-proline were purchased from Wako.

### Reagents and antibodies

Monoclonal mouse anti-p21 (1:200), polyclonal rabbit anti-PERK (phospho T981) (1:200), anti-PERK (1:200), and rabbit anti-ATF6α ﻿(1:200)﻿ antibodies were obtained from Santa Cruz Biotechnology (Santa Cruz, CA, USA); monoclonal mouse anti-CHOP antibody (1:1000) was obtained from Cell Signaling Technology (Danvers, MA, USA). Monoclonal rabbit anti-histone H3 (phospho S10) (1:1000), monoclonal rabbit anti-GCN2 (1:1000), and monoclonal rabbit anti-GCN2 (phospho T899) (1:500) antibodies were obtained from Abcam (Cambridge, UK). Monoclonal mouse anti-phospho-Histone H2A.X (Ser139) (1:50) antibody was purchased from Millipore (Billerica, MA, USA). Finally, polyclonal rabbit anti-actin (1:2000) antibody was obtained from Sigma-Aldrich (St. Louis, MO, USA).

### Analysis of cell viability, cell cycle progression, and apoptosis

Cells were seeded into 6-well plates at 100,000 cells per well. After 24 h, cultures were incubated with L-serine, D-serine, or both at the indicated concentrations for 48 h in the presence of 5% FCS. Cell number and viability were evaluated by using the Muse™ Count & Viability Assay Kit, cell cycle progression by using the Cell Cycle Assay Kit and apoptosis by using Annexin V staining, the Dead Cell Assay kit, and caspase 3/7 activity assays (components of the Muse™Cell Analyzer; Millipore) according to the manufacturer’s instructions.

### Cell proliferation assay using 3-(4,5-dimethylthiazol-2-yl)-5-(3-carboxymethoxyphenyl)- 2-(4-sulfophenyl)- 2H-tetrazolium, inner salt (MTS)

Cell viability was estimated by MTS assay using CellTiter 96 Aqueous One Solution reagent (Promega Corp., Madison, WI, USA) according to the manufacturer’s instructions. Briefly, cells were seeded into 96-well plates at 3,500 cells per well and incubated with L-serine, D-serine, or both in the presence of 5% FCS and the presence or absence of various drugs as indicated. Absorbance at 490 nm was measured on a microplate reader (EnSpire, Perkin-Elmer, Waltham, MA, USA) 1–3 h after adding 20 μL of the reagent to each well.

### Real-time quantitative and semi-quantitative PCR

Cell RNA was isolated by using RNAiso Plus (Takara, Shiga, Japan) and reverse transcribed by using PrimeScript RT Master Mix (Takara). Synthesized cDNA was used as a template for PCR quantification at 1:40 (vol/vol) in the PCR reaction mixture. PCR was performed on a CFX96 cycler (Bio-Rad, Hercules, CA, USA) with KAPA SYBR Fast Universal 2 qPCR Master Mix (Kapa Biosystems, Wilmington, MA, USA). Relative expression levels were calculated using β-actin mRNA expression as the reference. Primer sequences are listed in Table 1.

**Table 1:** Nucleotide sequences of the primers used for qRT-PCR in this study.

Gene	Primer Sequence
Human ß-actin	Forward 5′-TCCCCCAACTTGAGATGTATGAAG-3′
	Reverse 5′-AACTGGTCTCAAGTCAGTGTACAGG-3′
*BAX*	Forward 5′-TGGAGCTGCAGAGGATGATTG-3′
	Reverse 5′-CCCAGTTGAAGTTGCCGTCAG-3′
*BCL-2*	Forward 5′-TGGGAGAACAGGGTACGATA-3′
	Reverse 5′-CATCTCCCGCATCCCACTC-3′
*PUMA*	Forward 5′-GGTCCTCAGCCCTCGCTCTC-3′
	Reverse 5′-CTTGTCTCCGCCGCTCGTAC-3′
*P21*	Forward 5′-GTGGCCTTGTCGCTGTCTT-3′
	Reverse 5′-GCGCTTGGAGTGATAGAAATCTG-3′
*P16*	Forward 5′-GGGGGCACCAGAGGCAGT-3′
	Reverse 5′-GGTTGTGGCGGGGGCAGTT-3′
*IL-6*	Forward 5′-GGTACATCCTCGACGGCATCT-3′
	Reverse 5′-GTGCCTCTTTGCTGCTTTCAC-3′
*IL-8*	Forward 5′-AAGGAAAACTGGGTGCAGAG-3′
	Reverse 5′-ATTGCATCTGGCAACCCTAC-3′
*ATF4*	Forward 5′-GTTCTCCAGCGACAAGGCTA-3′
	Reverse 5′-ATCCTCCTTGCTGTTGTTGG-3′
*CHOP*	Forward 5′-TGCTTTCAGGTGTGGTGATGTA-3′
	Reverse 5′-AATCAGAGCTGGAACCTGAGGA-3′
*GCN2*	Forward 5′-GAAGCTGTCAGCCAGCACTA-3′
	Reverse 5′-GTTGGCAAGGGAGGTCTGAA-3′
*HK*	Forward 5′-GGCTCATTTCCACCTCACCA-3′
	Reverse 5′-GGAGGGCAGCATCTTAACCA-3′
*PFK*	Forward 5′-TGAAGCCAGAGAGGCCTTAGA-3′
	Reverse 5′- GGAACCAGGGAGAGATGTGC-3′
*GAPDH*	Forward 5′-CCTCAACGACCACTTTGTCA-3′
	Reverse 5′-TTACTCCTTGGAGGCCATGT-3′
*PGK1*	Forward 5′-CTGTGGCTTCTGGCATACCT-3′
	Reverse 5′-GCTGCTTTCAGGACCACAGT-3′
*PHGDH*	Forward 5′-ATCTCTCACGGGGGTTGTG-3′
	Reverse 5′-AGGCTCGCATCAGTGTCC-3′
*PSAT1*	Forward 5′-CGGTCCTGGAATACAAGGTG-3′
	Reverse 5′-AACCAAGCCCATGACGTAGA-3′

### Western blot analysis

Cultured cells were lysed by adding RIPA buffer (WAKO), and total protein concentration was measured by using a Pierce BCA Protein Assay Kit (ThermoFisher Scientific, Waltham, MA, USA). Sample buffer containing 0.25 M Tris-HCl (pH 6.8), 8% sodium dodecyl sulfate (SDS), 20% sucrose, 5% β-mercaptoethanol, and 0.02% bromophenol blue was added to the lysate and proteins were separated on 7.5–15% SDS polyacrylamide gels. Separated proteins were then transferred onto polyvinylidene difluoride transfer membranes (GE Healthcare, Buckinghamshire, UK) in a Tris-glycine transfer buffer (48 mM Tris-buffer, 39 mM glycine, 0.05% SDS, and 10% methanol). Membranes were incubated with primary and secondary antibodies as indicated, and an ECL Plus Western Blotting System (GE Healthcare) was used for detection. Reproducibility was confirmed in at least three independent experiments, and representative blots are presented in the figures. Band intensity was quantified using ImageJ (National Institutes of Health, Bethesda, MD, USA).

### Cytokine release

Human IL-6 and IL-8 levels were measured by chemiluminescent enzyme-linked immunosorbent assay (ELISA) (R&D Systems, Minneapolis, MN, USA) and homogeneous time resolved fluorescence (HTRF) (Cisbio Bioassays, Codolet, France), respectively, according to the manufacturers’ instructions.

### RNA interference

Gene expression was knocked down by using targeted siRNAs against human GCN2 (sc-45645, Santa Cruz), ATF4 (sc-35112, Santa Cruz), and CHOP (ThermoFisher Scientific, Cat. 1299001). Results were compared against a supplier-matched control siRNA (sc-37007 from Santa Cruz and Cat. 12935112 from ThermoFisher Scientific). These siRNAs were introduced into HK-2 cells using Screenfect A reagent (Wako) according to the manufacturer’s instructions.

### Statistical analysis

All data are reported as mean ± SEM. Two groups were compared by independent sample *t*-test and multiple groups by ANOVA with the post hoc Bonferroni tests for pair-wise comparisons. A *P* < 0.05 was considered statistically significant for all tests. GraphPad Prism software, version 6.00 for Macintosh (GraphPad Software, San Diego, CA, USA) was used for data analysis.

## Electronic supplementary material

Supplementary Information
