# Supplementary material for: D-serine, a novel uremic toxin, induces senescence in human renal tubular cells via GCN2 activation
Source: Sci Rep. 2017 Sep 11;7:11168. doi: 10.1038/s41598-017-11049-8 (PMC5593843; doi:10.1038/s41598-017-11049-8)

**D-serine, a novel uremic toxin, induces senescence in human renal tubular cells via GCN2 activation**

Akira Okada, Masaomi Nangaku, Tzu-Ming Jao, Hiroshi Maekawa, Yu Ishimono, Takahisa Kawakami, Reiko Inagi

**Supplementary Figure legends**

**Supplementary Figure 1**. DAO expression and effects of D-serine on cells other than human renal proximal tubules

(A) D-amino acid oxidase (DAO) expression in cDNA of HK-2, NHREC, HUVEC, and HepG2 cells. Only HepG2 cell expressed *DAO* after 35 cycles of PCR using *DAO* primers (forward: 5′-CGCAGACGTGATTGTCAACT-3′, reverse: 5′-GGATGATGTACGGGGAATTG-3′) or β-actin primers.

(B) Effects of D-serine in SV40Mes13, HUVEC, and HepG2 cells measured by MTS assay. Although D-serine affected cell viability in HK-2 cells and NHREC (see Fig. 1), but not in SV40 MES 13, HUVEC, or HepG2 cells. (n = 4 cultures per treatment group).


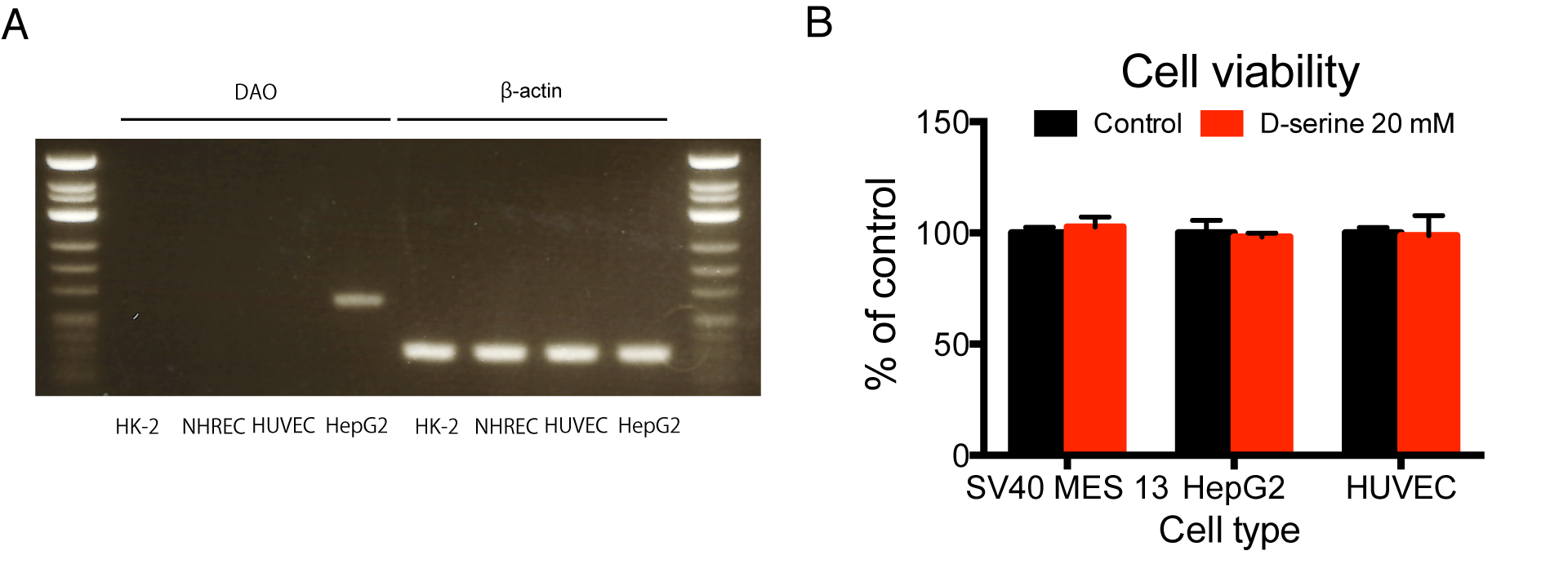


**Supplementary Figure 2.** Cell lysates were immunoblotted using anti-p21, anti-vimentin, anti-pGCN2, anti-GCN2, anti-CHOP, anti-pPERK, anti-PERK, anti-ATF6, and anti-actin antibodies. These cropped blots are shown in Fig. 2E, Fig. 4A, Fig. 4C and Fig. 4E.


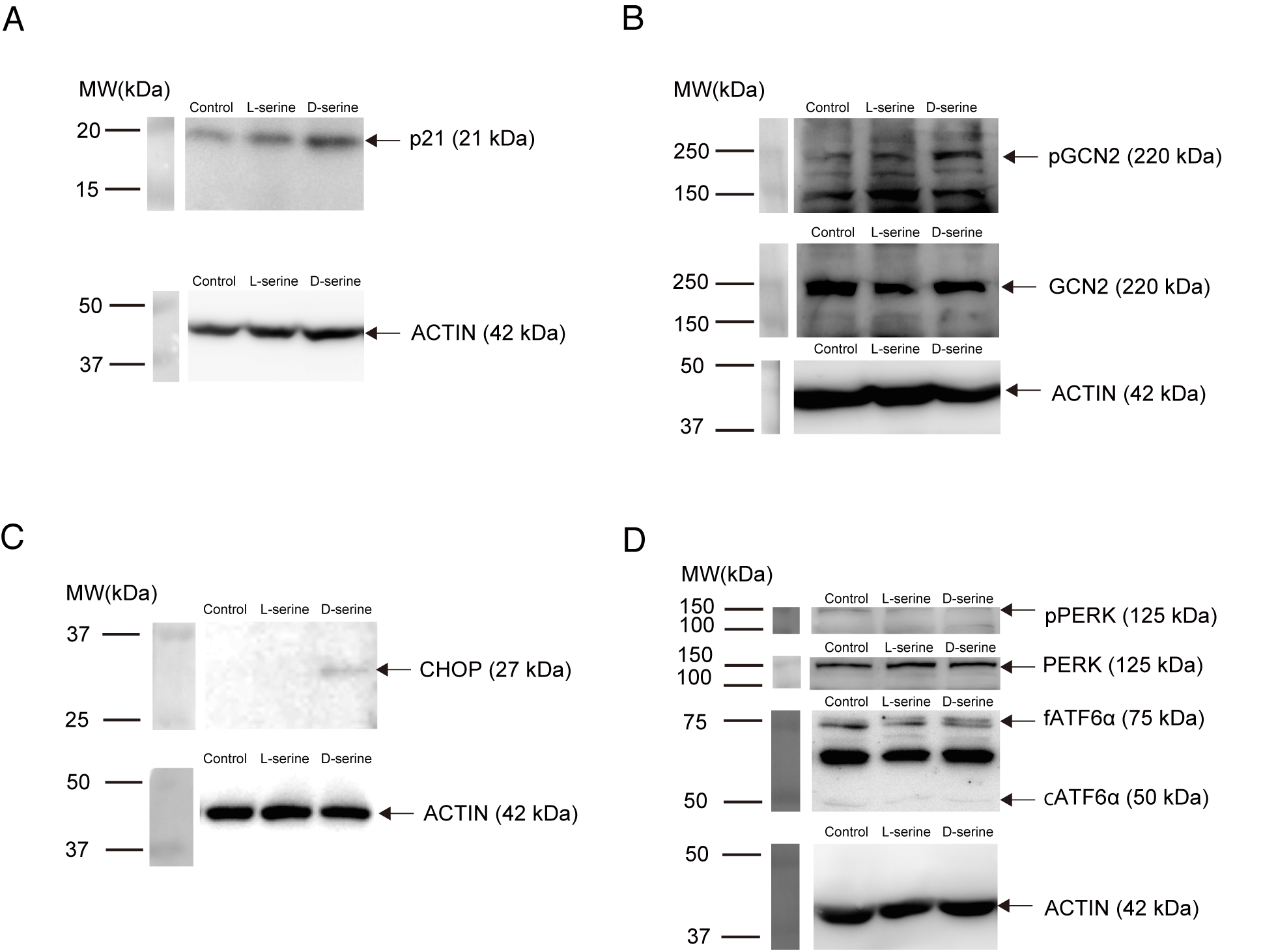

Supplement: Supplementary file 1 — Supplementary Information [file 41598_2017_11049_MOESM1_ESM.doc]
